# Molecular, Cellular and Functional Effects of Radiation-Induced Brain Injury: A Review

**DOI:** 10.3390/ijms161126068

**Published:** 2015-11-24

**Authors:** Sona Balentova, Marian Adamkov

**Affiliations:** Institute of Histology and Embryology, Jessenius Faculty of Medicine, Comenius University in Bratislava, Mala Hora 4, Martin 03601, Slovakia; balentova@jfmed.uniba.sk

**Keywords:** irradiation, radiation-induced brain injury, cognitive impairment, subventricular zone, hippocampus, interventional therapy

## Abstract

Radiation therapy is the most effective non-surgical treatment of primary brain tumors and metastases. Preclinical studies have provided valuable insights into pathogenesis of radiation-induced injury to the central nervous system. Radiation-induced brain injury can damage neuronal, glial and vascular compartments of the brain and may lead to molecular, cellular and functional changes. Given its central role in memory and adult neurogenesis, the majority of studies have focused on the hippocampus. These findings suggested that hippocampal avoidance in cranial radiotherapy prevents radiation-induced cognitive impairment of patients. However, multiple rodent studies have shown that this problem is more complex. As the radiation-induced cognitive impairment reflects hippocampal and non-hippocampal compartments, it is of critical importance to investigate molecular, cellular and functional modifications in various brain regions as well as their integration at clinically relevant doses and schedules. We here provide a literature overview, including our previously published results, in order to support the translation of preclinical findings to clinical practice, and improve the physical and mental status of patients with brain tumors.

## 1. Introduction

Preclinical studies have provided valuable knowledge about the pathogenic mechanisms involved in radiation-induced injury. During the past 10 years, several preclinical studies have demonstrated that interventional therapies that modulate neuroinflammation can prevent or ameliorate radiation-induced functional deficits. Translating these novel preclinical findings to clinical practice has the potential to improve the physical and mental status in patients with brain primary tumors and metastases.

Cognitive deficits, including progressive deficits in memory, attention and executive functions represent a significant risk for patients undergoing conventional radiotherapy. Cognitive impairment occur in 50%–90% of adult patients with brain tumors who survive more than six months after fractionated irradiation, frequently in the absence of corresponding anatomical abnormalities (e.g., white matter necrosis) [1]. Due to the improved radiotherapy treatment techniques, the patients with brain tumor survive longer but they experience the late effects of radiotherapy. Since the population of patients with late symptoms is growing rapidly, the current effort is focused on functional consequences of radiation the brain injury.

Based on the timeline and clinical expression, radiation-induced brain injury in clinical radiotherapy is divided into the three types: acute (during radiation up to days and weeks after irradiation), subacute or early-delayed (up to 12 weeks after irradiation) and late delayed (greater than six months to years after irradiation). The acute symptoms are characterized by drowsiness, headache, nausea, and vomiting as a result of increased intracranial pressure. The symptoms are mostly transient, reversible and may resolve spontaneously. Corticosteroids such as dexamethasone are sometimes needed to supress these symptoms. The subacute or early-delayed symptoms related to encephalopathy involve extreme somnolence, fatigue and deterioration of preexisting deficits that resolve within several months. Late radiation-induced changes are often progressive and irreversible. They are characterized by leukoencephalopathy syndrome, vascular abnormalities (*i.e.*, teleangiectasias, endothelial thickening, hyalinization, fibrinoid deposition, thrombosis and occlusion of vessels), true radionecrosis, brain parenchyma calcifications and increasing white matter abnormalities. The late effects include minor-to-severe neurocognitive deficits (e.g., decline in the hippocampal-dependent spatial learning and working memory, decreased verbal memory, intellectual decline, ataxia, urinary loss, dementia) [2].

On the cellular level, irradiation triggers a cascade of the direct and indirect effects including activation of early response transcription factors, cascades of signal transduction, alterations of proliferative vascular and glial cells, neurogenesis and neural functions. In this review we characterize the previous and recent outcomes of preclinical studies dealing with mechanisms or consequences of the radiation-induced brain injury and potential neuroprotective interventions.

## 2. Radiation-Induced Changes

### 2.1. Apoptosis

Irradiation of the brain most commonly damages DNA and consequently disrupts protein synthesis. There is also the specific effect of irradiation on certain metabolic pathways that have indirect effects on DNA transcription. Side effects of ionizing radiation on various metabolic pathways that are not directly involved in apoptosis occur even at very low doses (0.5 Gy) within hours after treatment [3]. Effects of acute or chronic irradiation include pertubations in ERK1/ERK2 (extracellular-signal-regulated kinase) and signaling pathways, that are crucial for neuronal survival after irradiation [3] and the activation of cell cycle checkpoints that are associated with increased Trp53 phosphorylation and Trp53 and p21 protein levels [4]. Single irradiation increases cytokines such as tumor necrosis factor-alpha; (TNFα), transforming growth factor beta 1 (TGF-β1) and several transcription factors (cAMP response element-binding protein; CREB, activator protein-1; AP-1, Sp-1, *etc.*) [5,6]. Interference with the cell cycle regulatory proteins and consequent apoptosis are the primary mechanisms responsible for cell death that occurs within several hours after treatment [4].

Numerous preclinical studies reported that the effect of ionizing radiation on apoptosis is dose-dependent and occurs within hours after treatment [7,8,9,10,11]. Radiation has been shown to induce apoptosis in the CNS (central nervous system), primarily in the neonatal or early postnatal brain but also in the brain of young adult rats. Irradiation with a single dose of 2 Gy caused the apoptosis of neurons and glial cells in the subvetricular zone (SVZ) covering the brain lateral ventricles (LV), olfactory bulb (OB), neocortex, pyriform and entorhinal cortex, dentate gyrus (DG), striatum, thalamus, amygdala, brain stem and the cerebral and cerebellar white matter [12]. Single whole-brain irradiation with large-scale doses (2–10 Gy) led to steep increases of apoptosis in the hippocampal DG within three to six hours after irradiation [7,8,9] and a plateau within six to 12 h after radiation treatment [10,11]. There was no additional increase of apoptosis, however the number of apoptotic cells remained unchanged within one to nine months postradiation [9,11,13,14,15]. Apoptosis was accompanied by a simultaneous reduction of proliferative 5-bromo-2′-deoxyuridine (BrdU) labelled cells, Ki-67 labelled cells and immature neurons labelled with doublecortin (DCX) (about 96% 48 h after irradiation, 60% three weeks later, 80% two months later) [8,11,13,16,17]. Results revealed that proliferating cells and immature neurons were impaired in a dose- and time-dependent mode. Surviving stem cells have limited potential of repopulation and regeneration of damaged self-renewing capacity several months after irradiation [9,15,17]. Reduction of hippocampal neurogenesis has been correlated with impairment in learning and spatial memory [13,14,18]. This fact is important from a medical perspective since the doses used in radiotherapy of brain tumors are usually much higher than the levels needed to eliminate neurogenesis [19]. However, a clear link between the radiation-induced cognitive impairment and inhibition of neurogenesis has not yet been demonstrated. Regarding the other neurogenic region, the SVZ, single (0.5–30 Gy) or fractionated (daily 1.5 Gy for 7 days) whole-brain irradiation led to the highest increase of apoptosis six hours after a single treatment followed by no additional apoptosis 48 h later [7,20]. Following fractionated irradiation, the first three fractions were effective at increasing apoptosis, however the rest of the fractions had no impact on the cellularity. Moreover, there was a significant rise in BrdU-labelled cells two to three days after irradiation, indicating increased cell proliferation. The proliferative response after depletion of cells via apoptosis may represent the recruitment of relatively quiescent stem/precursor cells [7,11]. Most recently a localized single irradiation of the right SVZ was performed followed by intracranial injection of lysolecithin, a substance that causes demyelinating lesions of the brain [21]. After the injection, irradiated SVZ displayed a higher proliferation rate and the SVZ-derived neuroblasts expanded from the irradiated SVZ toward the lesion site forming migratory chain-like structures. The injured hemisphere displayed an increase in the number of newly generated young oligodendrocytes that were incorporated into the demyelinated area and produced new myelin. These observations support the hypothesis that neural stem cells (NSCs) are radioresistant and can respond to a brain injury, recovering the neurogenic niche.

Little is known about side effects of irradiation. A dose-response loss of body and brain weight has been reported three weeks following single or fractionated irradiation which persists for at least 12 months but the cause of this change is not known [17,22,23,24,25]. One possible reason is the effect of irradiation on the olfactory system that could affect the sense of smell and apetite [26]. Previously published studies about head and neck irradiation of rats showed dysphagia with concomitant reduced body weight due to damage of salivary glands and pharynx [27], while another interpretation was reported in [28]. Fractionated irradiation of young and middle-aged rats with a total dose of 40 Gy delivered twice a week for four weeks revealed significant radiation-induced deficits in body, brain and pituitary weight, level of pituitary growth hormone (GH) and plasma insulin-like growth factor-I (IGF-I). These changes were greater in young rats than in rats irradiated in middle age and certainly contributed to decreased body weight and probably decreased brain weight. Taking into account that the GF/IGF-I axis modulates hippocampal neurogenesis and gliogenesis, radiation-induced changes in this system may influence cognitive and other neural functions.

### 2.2. Inflammatory Response and Oxidative Stress

Irradiation caused the oxidative stress as an outcome of a disturbace between production of reactive oxygen species (ROS) and antioxidant defense mechanisms. Irradiation activates microglia and causes infiltration of the brain. Upon activation, these cells produce ROS and activate more microglia and immune cells that can increase the level of oxidative stress. The common method for evaluation of oxidative stress is the measurement of inflammatory reaction to the increase of oxidative stress. Obviously, chronic oxidative stress is thought to result from an inflammatory response. Measurement of inflammatory response after *in vivo* and *in vitro* irradiation with various single doses (2–10 Gy) revealed increased expression of proinflammatory molecules such as TNFα, interleukin-1 beta; IL-1β, intercellular adhesion molecule-1; ICAM-1, cyclooxygenase 2; COX-2 [29,30,31,32], activation of transcription factors (AP-1, nuclear factor kappa B; NFκB, CREB) [30,32], and upregulation of mRNA levels of several chemokines (MCP1/CCL2, Gro/KC/CXCL1) [31]. Cranial irradiation with a single dose of ≥15 Gy resulted in acute infiltration of neutrophils and delayed increase in T cells, MHC (major histocompatibility complex) II-positive cells, and CD11c-positive cells at least one year after treatment [25]. Previous studies have been suggested that long-term microglial activation may be associated with a decrease of hippocampal neurogenesis and cognitive impairment [13,33,34]. Preclinical data promote a previous theory, that the late, radiation-induced injury is caused by oxidative stress and inflammatory response [35,36]. Administration of various anti-inflammmatory drugs prevents radiation-induced cognitive impairment (nonsteroidal and steroidal agents, COX inhibitors, *etc.*) [37,38,39].

### 2.3. Neurogenesis, Neurons and Neural Functions

The adult mammalian brain contains highly active sources of NSCs. Descendants of these multipotent neuronal progenitors, neuroblasts are produced in two discrete regions of the adult brain, the SVZ covering the brain LV and the subgranular zone (SGZ) of the hippocampal DG [40,41,42]. The DG-derived cells travel to the granular cell layer (GCL) and the progeny of the anterior SVZ (SVZa) traverse along the migratory route called the rostral migratory stream (RMS) to reach the olfactory bulb (OB). NSCs are capable of self-renewal as well as generating new neurons, astrocytes and oligodendrocytes [43,44,45].

**Figure 1:**
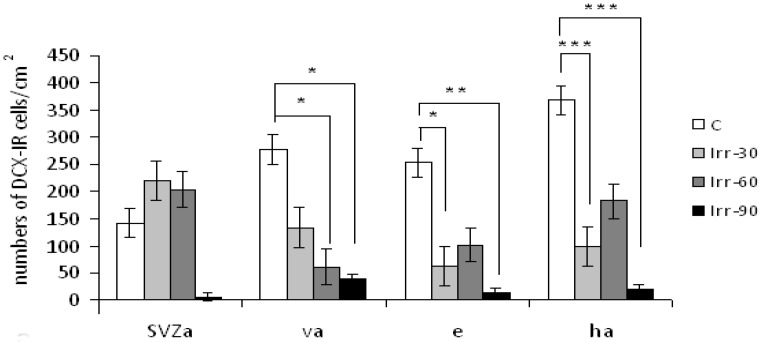
Distribution of DCX immunoreactive (DCX-IR) cells in the anterior horn of the subvetricular zone (SVZa), vertical arm (va), elbow (e), and horizontal arm (ha) in the RMS of control and irradiated adult male rats investigated 30, 60 and 90 days after fractionated irradiation with a total dose of 4 Gy (C, Irr-30, Irr-60, Irr-90; x ± SEM). Statistical significance of differences between control and irradiated group and between the irradiated animals: * *p* ≤ 0.05; ** *p* ≤ 0.01; *** *p* ≤ 0.001.

As a region of ongoing neurogenesis, the SGZ of the hippocampal DG is sensitive to therapeutic doses of radiation. Whole-brain single or fractionated irradiation of young adult mice and rats led to significant decrease of newborn mature and immature neurons in the DG and has been associated with decline in the hippocampal-dependent spatial learning and memory [13,14,33]. Although, older rats did not displayed radiation-induced decrease of neurogenesis, they exhibited cognitive impairment [46,47]. The hippocampal DG is not the only compartment that appears important in the radiation-induced cognitive impairment. Single or fractionated irradiation of rodent brain has been shown to result in a dose-dependent decrease of proliferation of surviving NSCs and differentiation of these cells into neurons in the SVZ of adult rats [7,20,22]. In our experimental studies we investigated the effect of fractionated irradiation (a total dose of 3–5 Gy, given as 1 Gy fractions once per week for three to five weeks) on neurogenic SVZ and subsequent regions represented the individual parts of the RMS, *i.e.*, the vertical arm, elbow and horizontal arm. In the brain of rats that survived 30–90 days after treatment, the SVZ displayed initial increase of DCX labelled cells and then the production of young neurons was slowing down (Figure 1) (Reproduced from [48] with the permission of Elsevier).

These results correspond with our findings regarding the radiation-induced initial increase and subsequent decline of young neurons throughthout the RMS until 60 days after treatment [49]. Our most recent study investigates the effect of a total dose of 20 Gy delivered once per week for four weeks on the SVZ and hippocampus. Our results confirm radiation-induced significant decrease of DCX-labelled young neurons up to 100 days after treatment is associated with a cognitive decline of hippocampal-dependent memory (unpublished results). This is in accordance with previous studies which, showed that irradiation led to massive elimination of DCX-labelled cells without concominant decrease in population of glial cells [11,13,23,47,50].

**Figure 2:**
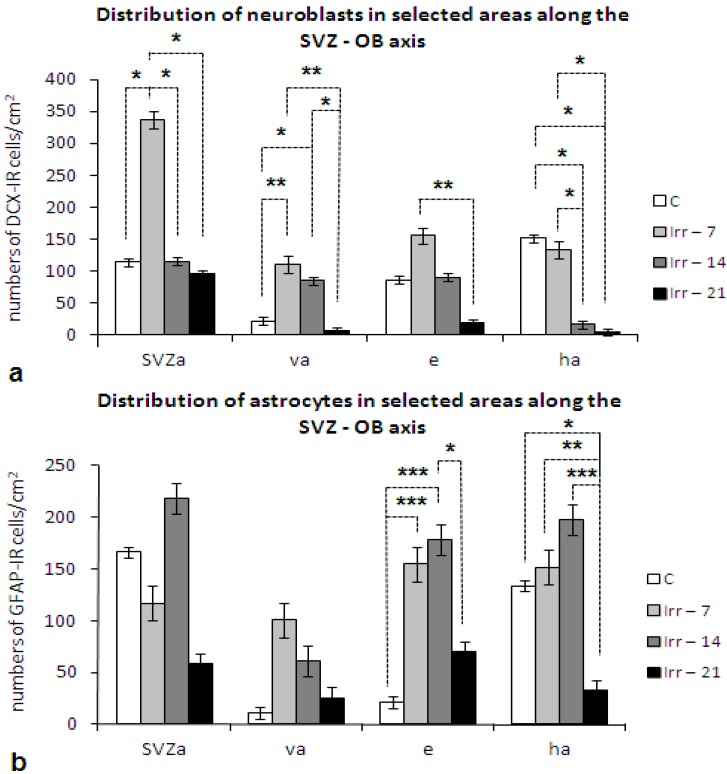
Distribution of (**a**) DCX immunoreactive (DCX-IR) and (**b**) GFAP (glial fibrillary acidic protein) immunoreactive (GFAP-IR) cells in the anterior horn of the subvetricular zone (SVZa), vertical arm (va), elbow (e), and horizontal arm (ha) in the RMS of control and irradiated adult male rats investigated at 7, 14 and 21 days after fractionated irradiation with a total dose of 3 Gy (C, Irr-7, Irr-14, Irr-21; $\bar{x}$ ± SEM). Statistical significance of differences between control and irradiated group and between the irradiated animals: * *p* ≤ 0.05; ** *p* ≤ 0.01; *** *p* ≤ 0.001.

In contrast, in the brain of rats that survived one to three weeks after irradiation, a prolonged increase of DCX labelled young neurons was seen 1 and 2 weeks in almost all parts of the RMS (Figure 2a).(Reproduced from [51] with the permission of Elsevier).

As mentioned above, increased cellularity may represent the recruitment of quiescent stem cells and this cellular input was seen after fractionated irradiation [7,11]. Preclinical studies confirmed, that application of a large single dose is more damaging than fractionated irradiation used in the conventional radiotherapy. The cell-mediated response is different; single exposure leads to steep reaction of the tissue and fractionated treatment cause delayed radiation-induced changes [52]. With a fractionated irradiation, the first dose kills the proliferating population and the cells are induced to undergo apoptosis. The mitotic activity is then restored and the next dose per fraction destroyed the cells that start to proliferate spontaneously or in response to the previous radiation-induced apoptosis [26]. The experimental studies revealed that there is a limit for a numbers of absorbed doses [7,17,23]. Parameters, which can influence the effect of radiation treatment include the dose rate, energy, activity and intensity of the source, source -to- axis distance (SAD), shielding*, etc.*

Previous studies suggested that partial brain irradiation did not cause the same degree of cognitive impairment as the whole-brain irradiation. Data from a clinical retrospective study of [53] that used neuroanatomical target theory showed that the incidence of radiation-induced cognitive decline depends rather on the dose to specific brain region than a total dose applied to whole brain. Thus, radiation-induced decline of neurogenesis may not predict radiation-induced cognitive impairment.

There is a growing interest in radiation-induced changes of neuronal function, predominatly synaptic plasticity and neuronal gene expression [54,55,56]. Irradiation produces changes in expression of the several immediate-early genes, such as activity-regulated cytoskeleton-associated protein (Arc), Homer1a, expression of glutamatergic *N*-methyl-d-aspartic acid (NMDA) receptor subunits, gamma-butyric acid (GABA) receptors, decrease in tyrosine phosphorylation, glutaminergic transmission and hippocampal long-term potentiation (LTP) [55,57,58,59]. These changes may occur without concomitant cellular changes following fractionated irradiation [60].

### 2.4. Glial Cells

Regarding the phenotypes of glial cells following irradiation, the oligodendrocytes required for fomation of myelin sheats did appear more radiosensitive than astrocytes. The key cells for production of mature oligodendrocytes are the progenitor cells, known as oligodendrocyte type-2 astrocytes (O-2A). Radiation-induced loss of O-2A leads to failure of their self-renewing capacity that ultimately results in demyelination and white matter necrosis. Irradiation of the rat cervical spinal cord with a single doses of 1–30 Gy examined until 24 h after treatment showed dramatic increase of oligodendroglial apoptosis and concomitant decrease of O-2A cells and mature oligodendrocytes [61,62]. In contrast to rat, O-2A cells in the mouse spinal cord appeared to be a radioresistant population. Single irradiation with a dose of 40 Gy led to less obvious decrease of O-2A cells (30%–35%) and their numbers remained constant with time. However, remaining surviving O-2A cells were functionally impaired *i.e.*, did not contribute to remyelination of axons [63]. Experiments done with p53 wild-type mice showed that radiation-induced apoptosis was preceded by an increase in nuclear p53 expression in glial cells of the spinal cord [64]. Results confirmed a clear dependence of radiation-induced apoptosis of oligodendrocytes on p53 protein. A similar radiation-induced oligodendroglial apoptosis was observed after whole-brain irradiation with doses of 10–22 Gy in the SVZ, and SGZ of the hippocampal DG and *corpus callosum* [10,64,65,66]. Several authors reported the increased numbers of O-2A cells [10,11] and this event did not reflect the production of new oligodendrocytes but rather represented a manifestation of radiation-induced inflammatory response [11]. In contrast, fractionated irradiation of rats with a total dose of 45 Gy and investigated one year later did not affect the number of oligodendrocytes, the size and number of myelinated axons, or thickness of myelin sheets, and these changes did not correspond to the observed cognitive impairment [67]. Thus, the relationship between radiation damage of oligodendrocytes and late radiation-induced changes remains unclear.

Astrocytes are the most numerous nonneuronal cell types in the CNS and represent about 50% of human brain volume. In the adult RMS, the astrocytes structurally support neurons and provide important signals and guidance for migrating young neurons toward the OB [42]. Many studies have reported an astrocytic response within the SVZ shortly after the radiation-induced loss of undifferentiated cells and neuroblasts [68,69]. Radiation has been reported to cause activation of astrocytes (reactive astrogliosis) and microglial cells at least six months after fractionated treatment [70,71]. Prominent features of the activation of astrocytes are proliferation, hypertrophy of cell body and processes, upregulation of intermediate filaments (GFAP), secretion of a host of proinflammatory mediators (COX, ICAM-1) and many other factors that mediate inflammatory and remodeling processes [29,72]. These changes involve alterations in gene expression and cell hypertrophy and persisting glial scar formation. There is growing evidence that reactive astrogliosis play either fundamental or contributing roles in CNS disorders. Increased astrogliosis with concomitant cognitive dysfunction were described in experimental model of acute encephalopathy [73]. Single irradiation with lower doses (>8 Gy) did not affect the numbers of GFAP-expressing astrocytes, but higher doses (20–45 Gy) led to increased astrogliosis and persisted one year after radiation delivery [74,75,76].

**Figure 3:**
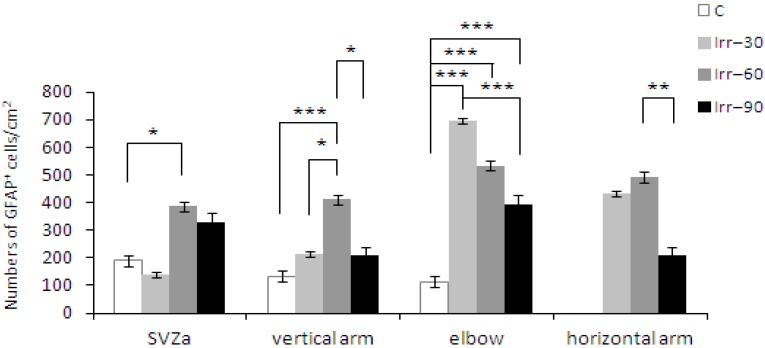
Distribution of astrocytes in individual parts along the SVZ-OB axis in the forebrain of control animals and rats, survived 30, 60 and 90 days after fractionated radiation treatment with a total dose of 4 Gy (C, Irr-30, Irr-60, Irr-90; $\bar{x}$ ± SEM). Statistical significance of differences between control and irradiated groups and between the irradiated animals: * *p* ≤ 0.05; ** *p* ≤ 0.01; *** *p* ≤ 0.001.

Despite different dose fractions and survival times, our experimental studies in rats revealed a clear astrocytic response [51,77,78]. Fractionated irradiation with a total dose of 3 Gy (1 Gy/d, 1 d/week for 3 weeks) resulted in a significant enhancement of the GFAP-labelled astrocytes in the RMS two weeks after treatment. However, this increase was only temporary and in the next week was replaced by a significant decline [51] (Figure 2b). Following irradiation with a total dose of 4 Gy (1 Gy/d, 1 d/week for 4 weeks), a significant increase of astrocytes was dominated 60 days after treatment, but in the end of the experiment the numbers returned back to control values (Figure 3). (Reproduced from [78] with the permission of the journal General Physiology and Biophysics).

Most recently, whole-brain irradiation with a total dose of 20 Gy (5 Gy/d, 1 d/week for four weeks) confirmed our previous finding and a significant astrocytic response was seen 100 days after treatment (unpublished results).

Single or fractionated exposure (total doses of 20 or 40 Gy) led to changes in endothelial cell adhesion, blood-brain barrier (BBB) permeability and reactive astrogliosis accompanied with cognitive impairment [71,73,75]. Although radiation-induced astrogliosis is not directly a marker of inflammation, it is associated with or is a byproduct of neuroinflammation. *In vitro* experiments with microglia conditioned medium revealed that radiation-induced astrogliosis is caused by the factors released from irradiated microglia, such as mRNA levels for COX-2, IL1-β, interleukin 6; IL-6, interleukin 18; IL-18, TNF-α, and interferon-gamma-inducible protein-10; IP-10 [79].

Microglial cells are the resident mononuclear phagocytes of the CNS involved in the maintenance of brain homeostasis and immune defense. They have remarkable functional plasticity and capacity to expand in response to injury and acute or chronic diseases in the CNS [80,81]. Microglia become activated after injury and this process involves morphological transformation, and increased expression of the proinflammatory genes (COX, IL1-β, IL-6, TNF-α) [32,79]. Dysregulation of microglial activation and microglia-induced inflammation is detectable in virtually all brain pathological events. Single *in vivo* or *in vitro* irradiation led to increase in the expression of proinflammatory mediators (ICAM, TNF-α, IL1-β), apoptosis-related, stem cell-related, trophic and transcription factors and several surface molecules such as ionized calcium binding adaptor molecule (Iba1), lectin binding sugar molecules, enzyme nucleoside diphosphatase (NDPase) or cluster of differentiation molecule 11b (CD11b) [82]. Rodent studies also detected the increase of activated microglia in the brain during the latent period before expression of late radiation-induced injury [83,84].

In our experiments, there was a clear impact of fractionated irradiation on the population of resting microglia expressing the CD11b antigen. Irradiation with a total dose of 4 or 5 Gy (1 Gy/d, 1 d/week for 4 or 5 weeks) led to a significant decline or absence of microglia until 90 days after treatment and a non-expected decline or absence of activated microglia expressing CD68 antigen [48,49]. However, results obtained from other laboratories reported a clear radiation-induced increase of activated microglia [11,47,50]. A possible interpretation of these findings provides a technique used for radiation delivery. Whole-body irradiation did not initiate as intense radiation response as the whole-brain irradiation. Another reason for a weak CD68 immunoreactivity is that the CD11b marker is co-expressed in resting as well as activated forms of microglia. Given the role of proinflammatory mediators in radiation injury, it is possible that the activation of microglia may constitute a critical factor in the radiation-induced inhibition of neurogenesis [11]. Nowadays, several therapeutic approaches prevent or mitigate radiation-induced cognitive impairment through the administration of anti-inflammatory agents, or orthotopic injection of neuronal stem cells [85,86,87,88].

### 2.5. Endothelial Cells

Numerous studies provide evidence of radiation-induced structural changes, represented by loss of endothelial cells, enlargement of endothelial cell nuclei, vessel dilatation, vessel wall thickening, decrease in the vessel density and length, increase in vessel permeability accompanied by an increase in leukocytes adhesion in pial vessels and altered integrity of endothelial tight junctions [71,89,90,91,92,93,94,95]. Radiation-induced injury may lead to the production of free radicals under hypoxic conditions. Profound vascular rarefaction and hypoxia was found in the hippocampus two months after irradiation [96]. In contrast, chronic systemic hypoxia following irradiation led to restoration of the microvascular density [97]. On the other hand, tissue oxygen conditions could be improved by hyperbaric oxygen treatment (HBO). Several clinical reports described successful treatment of late CNS toxicity by prophylactic HBO [98,99,100]. However, reoxygenation may paradoxically accelerate axonal injury [101]. Hypoxia is also a crucial stimulus for increase of vascular endothelial growth factor (VEGF) expression, which is known to mediate increased vascular permeability [102]. A model of rat myelopathy after single irradiation with doses of 8 to 22 Gy revealed that the upregulation of VEGF expression in astrocytes is associated with a decrease of vascular permeability without a concomitant endothelial proliferation [103]. Functional consequences of altered VEGF expressions were examined on transgenic mice with reduced VEGF. Following irradiation to the thoracolumbar spinal cord, transgenic mice displayed longer time to development of paralysis compared to wild-type mice suggesting that the VEGF inhibition may cause protection [104]. The importance of the vascular integrity is well-documented by a model of radiation-induced myelopathy. Boron-neutron capture therapy displayed that the remainder of clonogenic O-2A cells after treatment was significantly higher when the radiation dose was primarily delivered to the vascular endothelium [105].

Ionizing radiation induced the early endothelial cell apoptosis within 24 h after the single whole-brain or spinal cord irradiation [89,93,106,107]. The radiation-induced apoptosis of endothelial cells is mediated by the lipid second messenger ceramide via activation of acid sphingomyelinase (ASM). Genetic model of inherited deficiency of ASM activity displayed reduction of the endothelial cell apoptosis [106,107,108]. Inhibiting ASM activity might provide a highly specific approach to reduce endothelial cell apoptosis [109]. Another protection against the apoptotic endothelial cell death represents administration of various growth factors [106,110,111].

As reported in many studies, endothelial cell apoptosis initiates acute BBB disruption. Experiments made with administration of various exogenous BBB permeability tracers showed that changes of BBB permeability are dose- and time-dependent, and often depend on molecular weight and the corresponding permeability of tracers [92,93,112]. Single irradiation with doses of 2 to 50 Gy leads to the acute increase of BBB permeability that recovers over several weeks [92,93]. In contrast, fractionated irradiation with a total dose of 40 Gy delivered five days per week for four weeks results in long-lasting increase of BBB permeability several months after treatment [71,113]. Long-lasting increases of BBB permeability corresponds to the late radiation-induced disruption of BBB and this event precedes gross-white matter damage. It is not clear whether the early endothelial apoptosis contribute to the late vessel density changes and BBB disruption. In contrast, radiation-induced white matter necrosis has been shown to occur in absence of vascular changes [90].

Althought the vascular injury is recognized as a primary cause of radiation-induced changes, the pathophysiology of late injury is multifactorial (e.g., demyelinisation, microvascular changes, decline of neurogenesis, glial cells proliferation or decline) [114].

### 2.6. Neurocognitive Functions

Preclinical studies provided valuable knowledge about the pathogenic mechanisms involved in the radiation-induced cognitive dysfunction. Regarding the crucial role of the hippocampus in the consolidation of information from short-term to long-term memory, spatial navigation and learning, most rodent studies have focused on the hippocampus. Several studies reported that reduction of hippocampal neurogenesis has been correlated with spatial memory and learning deficits [14,18,75]. Irradiation of the young adult rodents led to impaired performance in the Morris water maze (MWM) [14,75,115], passive avoidance [75,116], Barnes maze [13,117], novel object recognition [50,115] and T-maze tasks [18]. On the contrary, another study [17] showed that the radiation-induced decline of neurogenesis is not accompanied by impairment in non-matching sample task (NMTS), which measured conditional rule learning and memory for specific events and the fear conditioning task, to examine long-lasting associative learning. Moreover, previous studies showed that radiation-induced injury is significantly influenced by age. The radiation-induced decline of neurogenesis that appears to contribute to the cognitive deficits in young rodents does not occur in old rats [46,47]. In contrast, older rats exhibit greater inflammatory response that may play a role in the development of neural dysfunction [47,55].

As previously mentioned, the hippocampus is not the only compartment that appears important in radiation-induced cognitive impairment. Significant impairment of the perirhinal cortex-dependent function was found from six to seven months after fractionated irradiation with a total dose of 40 Gy delivered twice a week for four weeks. Moreover, the fractionated irradiation failed to alter hippocampal-dependent cognitive function, despite a significant reduction in the hippocampal neurogenesis and radiation-induced increase in activated microglia [50,115]. These findings confirmed that irradiation leads to hippocampal- and non-hippocampal dependent cognitive impairment in multiple brain regions.

Little is known about relevance of cellular input of new neurons derived from the SVZ and migrated via RMS towards the OB. Several studies revealed that new neurons are not necessary for olfaction, and others declared association between neurogenesis and olfactory functions [118,119,120]. For instance, investigation of the odor detection, discrimination and olfactory memory after single irradiation of SVZ confirmed that production of new neurons is not required for any of the tested olfactory functions [121]. Regarding the translation of preclinical data to clinical practise, impairment of olfaction may precede Parkinson’s disease in otherwise asymptomatic olfactory function [122,123]. An experimental model of Parkinson’s disease revealed a decrease of the odor discrimination and concominant reduction of neurogenesis [124]. The purpose of the upcoming preclinical study is enhancement of neurogenesis in the senescent or neurodegenerative brain and recovery of brain functions [125,126].

## 3. Preclinical Approaches to Preserve/Mitigate Radiation Injury

To prevent and ameliorate radiation-induced changes, numerous preclinical concepts were investigated under experimental conditions. Potential therapeutic interventions against radiation-induced injury were based on different approaches: (a) reducing apoptosis by e.g., inhibition of ASM activity [109], inhibition of VEGF, administration of basic fibroblast growth factor (bFGF) [106], platelet-derived growth factor (PDGF), insulin-like growth factor-1 (IGF-1) [110,111]; (b) inhibition of inflammatory response by nonsteroidal and steroidal agents, COX inhibitors, PPAR (peroxisomal proliferator-activated receptor) agonists [37,38,39]; (c) oxygen starvation or HBO [97,98]; (d) administration of EPO [127]; (e) RAS blockers [50,128]; (f) stem cell therapy [87,129]; and (g) enviromental enrichment [130,131], *etc.* Regarding the growth factors, preclinical studies showed that they can increase the long-term radiation tolerance of the spinal cord [110,111,132]. Moreover, the VEGF pathway inhibition with bevacizumab might be able to reduce the magnetic resonance imaging (MRI) abnormalities associated with necrosis [133]. However, application of the right dose, timing and combination of various growth factors seem to be problematic for human radiotherapy.

Another important potential neuroprotective agent is erythropoietin (EPO). The neuroprotective effect of EPO was confirmed in a variety of experimental brain injuries (e.g., ischemia, concussive brain injury, experimental autoimmune encephalomyelitis, and kainate-induced neurotoxicity) [134,135,136]. EPO can impact the progression of disorders such as Alzheimer’s disease, Parkinson’s disease, retinal injury, stroke, and demyelinating disease [137]. EPO has been used extensively over the last 20 or 25 years for the treatment of anemia in cancer patients. Therefore, neuroprotective effects observed in preclinical models can be easily translated to the clinic. In rodent studies EPO administration before or after single irradiation protects against the motor impairment and deficits in hippocampal-dependent learning and memory [127]. Recently, EPO has been not validated for post-radiation therapy because previous clinical trials revealed potential adverse effect of EPO on tumor control [138].

Most novel potentional therapeutic strategies have focused on anti-inflammatory drugs and transplantation of neural stem cells. Several rodent studies are focus on drugs that have been used succesfully for many years in clinical practice to treat other symptoms [139,140]. The PPARα, δ and γ agonists are the anti-inflammatory agents that have been investigated in preclinical studies. PPAR agonists regulate inflammatory signaling and act as neuroprotectants in various CNS diseases [141,142]. A model of PPARδ knockout mice with dietary administration of PPARδ agonist, GW0742 sucessfully inhibited neuroinflammation but did not restore neurogenesis or prevent the early-delayed cognitive impairment [117]. The PPARγ agonist pioglitazone (Pio) has been prescribed for several years as an antidiabetic agent. Animal studies showed that administration of Pio before or during irradiation with a total dose of 40 Gy prevented the radiation-induced cognitive impairment up to 54 weeks after the treatment. In contrast, therapeutic intervention with Pio after the completion of radiation treatment reduced the radiation-induced change only substantially [36]. Recently, a phase I/II clinical trial has been initiated to determine the dose of pioglitazone that can be given safely to brain tumor patients at Wake Forest Baptist Medical Center, Winston-Salem, NC, USA.

A more recent study [115] reported that dietary administration of the PPARα agonist, fenofibrate, has radioprotective effects. Continuous administration of fenofibrate one week prior to radiation treatment until 30 weeks afterwards, prevented the radiation-induced impairment in the perirhinal cortex, but failed to alter the hippocampal-dependent cognitive fuction and neurogenesis. On the other hand, a mouse model of treatment with identical doses of fenofibrate prior to the single irradiation (a dose of 10 Gy) preserved the hippocampal neurogenesis by promoting the survival of newborn cells and inhibited microglial activation [143]. Different findings may be due to species-specific responses to irradiation or are associated with a different radiation response of the brain to single and fractionated irradiation. The potential therapeutic effect of fenofibrate on radiation-induced changes in cognitive tasks suggests that it may be clinically beneficial.

Another promising therapeutic approach in the prevention and treatment of late radiation-induced changes is a blockade of the renin-angiotensin system (RAS). Angiotensin-converting enzyme inhibitors (ACEI) or angiotensin type II receptor blockers (ARB) are routinely prescribed for clinical treatment of hypertension or cardiovascular disease and have been succesfully proven in the experimental model of nephropathy [144] and pneumopathy [145]. To assess the effects of RAS blockers on radiation-induced brain injury, previous preclinical studies have been focused on the optic nerve, one of the most critical and radiosensitive structures in the brain. A model of optic neuropathy in the rat revealed that irradiation with a single dose of 30 Gy combined with chronic administration of ACEI, ramipril can mitigate the radiation-induced-induced optic nerve damage and preserve the functional integrity of the nerve at least six months after irradiation [146,147]. Chronic administration of ramipril beginning 24 h after single irradiation with single doses of 10 or 15 Gy may preserve neurogenesis but does not protect against neuroinflammation [128]. In contrast, a more recent study [50] has shown that combination of fractionated irradiation with a total dose of 40 Gy delivered twice a week for four weeks and administration of ramipril before, during and after irradiation prevented the perirhinal cortex-dependent cognitive dysfuntion and activation of microglia but did not preserve neurogenesis. These findings raise a question about the timing of ramipril administration and different dose response after the single and fractionated irradiation. A phase I/II of clinical trial is being developed to determine if ramipril and also ARB can prevent or mitigate the radiation-induced cognitive impairment in brain tumor patients.

Simultaneously with pharmaceutical intervention, there is growing interest in the use of various sources of stem cells. Transplantation of NSCs has been considered as an effective therapeutic strategy in a variety of neurological disorders characterized by the collapse CNS repair mechanisms in restoring the tissue damage and rescuing the lost function. Cellular sources for NSCs include fetal and adult CNS-derived NSCs, neural progenitors and a wide range of non-neural stem cells such as mesenchymal stem cells (MSCs) and hematopoietic stem cells (HSCs). Given that the function of surviving NSCs may be adversely affected by irradiation, there is a strong rationale for pursuing transplantation-based stem cell strategies for improving cognition after cranial irradiation.

Numerous preclinical studies have been focused on xenogenic transplantation of human pluripotent NSCs into the rodent host [87,129,148]. Irradiation of rats followed two days later by intrahippocampal transplantation with human NSCs ameliorates hippocampal-dependent cognitive impairment and preserves neurogenesis [87,148]. A recent study [129] was conducted on the intracranial transplantation of human ESC-derived oligodendrocytes four weeks after fractionated irradiation with a clinically relevant dose. Behavioral testing showed a complete recovery of cognitive function 10 weeks after grafting while additional recovery from motor deficits required concomitant transplantation into the cerebellum.

Preclinical studies with stem cell transplantation has previously led to numerous clinical applications [149,150]. However, this approach has to be qualified in the context of potential problems including immune rejection [151], teratogenesis and tumorigenesis [152], and ethical principles [153]. Besides the stem cell-based studies, enviromental enrichment has been shown to have a positive impact on the brain function not only in healthy animals but also in those with traumatic brain injury, stroke, epilepsy, Parkinson’s disease, and Huntington disease, and the functional improvement is partially mediated through the enhancement of neurogenesis [154,155,156,157]. Generally, voluntary physical activity has been a very robust stimulus for adult hippocampal neurogenesis in rodents from birth to oldest age [158,159,160]. For instance, gerbils exposed to single (5–10 Gy) irradiation following rotorod memory learning displayed increased numbers of newborn neurons and significant improvement of the spatial learning and memory two months later [15]. Further studies with irradiated mice showed that voluntary wheel running increases hippocampal neurogenesis and prevents progressive memory decline [130,131]. A clinical trial of aerobic exercise in children treated with cranial radiation for brain tumours to promote the hippocampal neurogenesis is ongoing at the Hospital for Sick Children, University of Toronto, Canada.

Based on the most recent preclinical findings, it is highly optimistic to think that one approach will eliminate every consequences of the radiation-induced brain injury. However, the use of stem cell based therapies or pharmaceutical interventions are promising and require more research before they can be translated into the clinical practice.

## 4. Conclusions

Preclinical studies are valuable model studies that provide significant findings about the mechanisms and consequences of radiation-induced injury. Modern radiotherapeutic techniques have eliminated acute and early-delayed brain injury as well as the late demyelination and white matter necrosis. On the other hand, prevention or amelioration of cognitive impairment is still problematic. Regarding the preclinical findings, the most effective treatments have to be given prior to, during and continuously after irradiation. Early clinical trials have only modest success in modulating radiation-induced cognitive impairment; however, results from the most recent studies look promising.
